# Supplementary material for: The interactome of intact mitochondria by cross-linking mass spectrometry provides evidence for coexisting respiratory supercomplexes
Source: Mol Cell Proteomics. 2017 Dec 8;17(2):216–32. doi: 10.1074/mcp.RA117.000470 (PMC5795388; doi:10.1074/mcp.RA117.000470)
Supplement: Supplemental Data [file supp_RA117.000470_133922_0_supp_23978_fzffwf.zip › spectra_annotation/mito_DR_spectra_annotation/448-1-5-1-4-1.pdf]

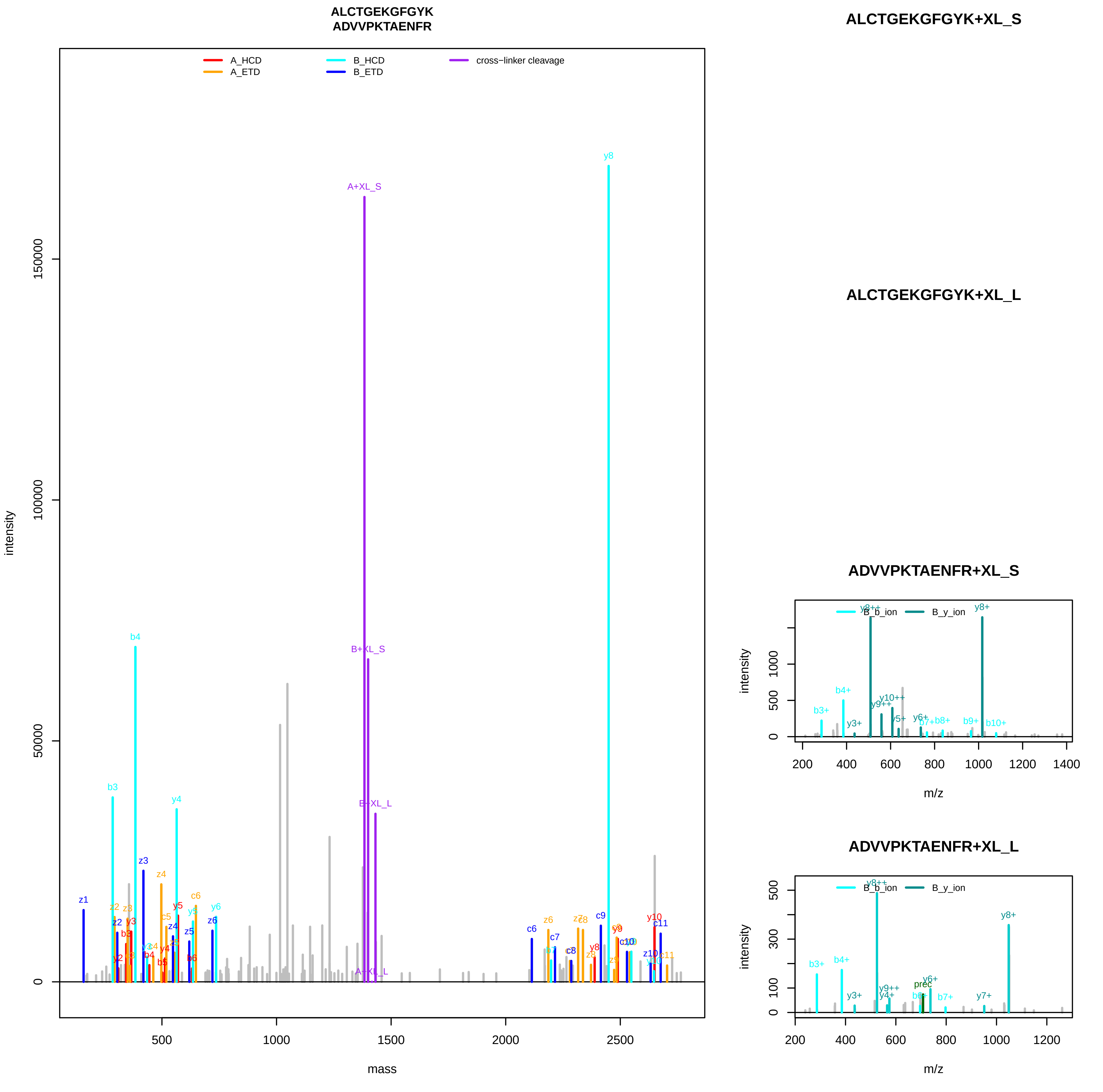
ALCTGEKGFGYK
ADVVPKTAENFR
A_HCD
A_ETD
B_HCD
B_ETD
cross−linker cleavage
intensity
mass
0
50000
100000
150000
500
1000
1500
2000
2500
y8
A+XL_S
B+XL_S
B+XL_L
A+XL_L
b4
b3
y4
z3
z4
z1
c6
y6
z2
z3
y3
y5
b3
z2
z4
z5
z6
b4
y2
c5
c6
c7
c8
c9
y8
y10
c11
c10
z10
y9
z7
c8
ALCTGEKGFGYK+XL_S
ALCTGEKGFGYK+XL_L
ADVVPKTAENFR+XL_S
B_b_ion
B_y_ion
y8++
y8+
b4+
b3+
y3+
y9++
y10++
y5+
y6+
b7+
b8+
b9+
b10+
m/z
200
400
600
800
1000
1200
1400
0
500
1000
ADVVPKTAENFR+XL_L
y8++
y8+
b3+
b4+
y3+
y9++
y4+
prec
y6+
b6+
b7+
y7+
0
100
300
500
